# Making WHO recommendations more responsive

**DOI:** 10.2471/BLT.14.021114

**Published:** 2014-11-01

## Abstract

Recent World Health Organization (WHO) guidelines not only advise countries on what treatment to give patients and when, but also how to roll this out in countries. Priya Shetty reports.

Just a decade ago few patients in developing countries were receiving antiretroviral (ARV) medicines; HIV infection was in most cases a death sentence. In 2003, the 3 by 5 campaign was launched to extend this life-saving treatment to those in need.

With nearly 10 million HIV-positive people receiving treatment in developing countries at the end of 2012, the global roll-out of ARVs has been a major public health success – one in which clinical guidelines play an important role. In South Africa, for example, adult life expectancy has increased by 11 years since 2004, when treatment was first provided by the public health system.

**Figure:**
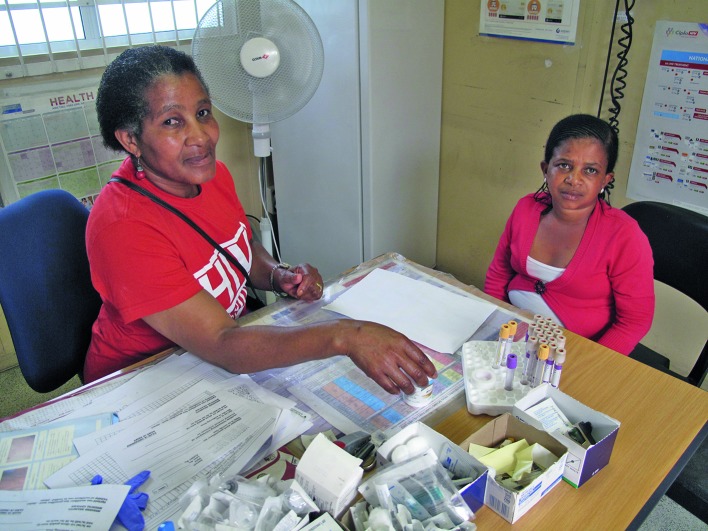
A nurse shows a community health worker the drugs used to treat children for HIV infection, including syrups, at the Ubuntu Clinic in Khayelitsha, Cape Town, South Africa

WHO/T Namjilsuren (2013)

But, over the years, HIV programme managers started to feel overwhelmed. “The message we were getting was that WHO was generating so many guidelines, technical updates, programmatic updates and so on that it was quite challenging to implement them,” says Dr Philippa Easterbrook from WHO’s HIV department.

“The idea was to create a one-stop shop,” says Easterbrook, “which would unite existing advice with new guidance to provide a complete view of all the current information on HIV treatment.” So far, the feedback from countries has been positive, she says, and as a result, a similar approach is being pursued with other HIV department guidelines, including plans for consolidated guidelines on: strategic information, HIV testing and hepatitis C screening, care and treatment.

“The idea was to create a one-stop shop.”

Philippa Easterbrook

In 2013, WHO issued the *Consolidated guidelines on the use of antiretroviral drugs for treating and preventing HIV infection: recommendations for a public health approach*, including a new recommendation to initiate treatment at an earlier stage of the infection – thus increasing the number of people globally who would be eligible for treatment. 

The WHO recommendation change was based on evidence accumulated over the years showing that early initiation of treatment allows people with HIV infection to live longer, healthier lives while reducing the risk of infecting others. And it was estimated that such a change could avert an additional 3 million deaths and prevent 3.5 million more new HIV infections by 2025. But increasing the pool of people who are eligible for ARV treatment poses a major challenge for countries, like South Africa, facing health worker shortages and other health systems problems.

Despite that, Dr Yogan Pillay, Deputy Director General, HIV/AIDS, Tuberculosis and Maternal Child and Women’s Health at the National Department of Health in South Africa, says the consolidated HIV guidelines were “very well received” not least because these were accompanied by advice on how to deliver these services and, of course, the ultimate goal is to save even more lives. “The regional workshops on service delivery that were held after the guidelines were launched were very useful,” Pillay says.

The global health landscape has changed considerably since WHO was founded in 1948 and started issuing guidance on a wide range of health areas to its Member States. But, while its mandate to “make recommendations with respect to international health matters” is anchored in its Constitution, the process for developing recommendations has evolved over the years.

The most recent shift in WHO’s approach to making recommendations came after an external review of the guideline development process in 2007. The review concluded that WHO guidelines drew too heavily on expert opinion, and not enough on systematic reviews of evidence, and that there was a lack of transparency about the way they were developed. There was also a question over the extent to which they were driven by public health need, says Dr Charles Penn, who is currently the chair of the WHO Guidelines Review Committee that was set up in 2008. 

Since then, WHO has put in place a set of standards for developing guidelines, which are constantly evolving as methods in the field change, to ensure that they reflect the best available scientific evidence, a shift that the Guidelines Review Committee oversees.

A key feature of this new emphasis on systematic use of evidence has been the adoption of the grading of recommendations, assessment, development and evaluations methods, best known by its acronym GRADE, to assess the quality of evidence and strength of recommendations based on this evidence.

The idea, says Penn, is to separate evidence retrieval from expert analysis, “so that expert panels are looking at the evidence put in front of them, and not coming with their own preconceptions and biases”.

But the quality of evidence is not the same in every field, which is a challenge for those intent on producing strong, evidence-based guidelines. “It is often easiest to deal with clinical management of disease, especially pharmaceutical interventions. This is an area of health where you have the highest quality of evidence because you can do randomized controlled trials, which score highly in GRADE,” Penn says.

In addition, qualitative research – or research on issues for which it is not possible to conduct randomized controlled trials – is often considered to be of low quality, which some researchers can take issue with, says Penn. But this is an evolving process, he says. “WHO is constantly looking at how to strengthen the GRADE methods as well as ways in which other types of evidence can be assessed and the applicability of GRADE can be broadened.”

Once the guidelines have been developed at the global or regional level, WHO then needs to assist countries in adapting guidelines to their context. The consolidated HIV treatment guidelines are a case in point.

For Dr Peter Cherutich, director of HIV prevention at the National AIDS & Sexually Transmitted Infections Control Programme at Kenya’s Ministry of Health, whose job it is to implement HIV guidelines, the new set of harmonized HIV treatment guidelines were useful – not least because they took into account the situation on the ground in the countries that were most likely to use them.

“WHO has done an excellent job in guiding countries. The challenge has been the complexity of local considerations, in terms of cost, training needs and the diversity of local stakeholders – since the engagement of civil society in the implementation of clinical guidelines is still a new concept in Kenya – and the sheer time it takes to cascade the guidelines to lower level facilities,” Cherutich says. “WHO has a clear framework for doing this and has been able to mobilize other partners to assist Kenya to roll them out.”

WHO guidelines can often be a catalyst in driving a policy change that a country is already contemplating, says Easterbrook, who co-led the development of the consolidated HIV guidelines.

At the same time, when WHO issues new guidance, some countries may only just be implementing policy from the previous set of guidance.

This can prove problematic at the treatment level, says Dr Halima Dawood, head of infectious diseases at Greys Hospital in KwaZulu-Natal, South Africa, which provides tertiary care and clinical support to HIV treatment clinics in western KwaZulu-Natal. “In South Africa, WHO guidance can be difficult to implement if it has not been sanctioned by the local department of health.”

“WHO guidance can be difficult to implement if it has not been sanctioned by the local department of health.”

Halima Dawood

“WHO needs to better monitor the implementation of guidelines, to find out what works and what doesn’t, before proposing new guidelines,” agrees Pillay. “While science must lead, the issue of capacity to implement new guidelines is also important.”

Input on the feasibility of guidance has long been incorporated during the guidelines development process. But the consolidated HIV treatment guidelines were different, as they draw on input from 120 stakeholders – including clinicians, researchers, implementers and community representatives – and more than 100 peer reviewers. “We collated thousands of comments on the guidelines,” says Easterbrook.

**Figure:**
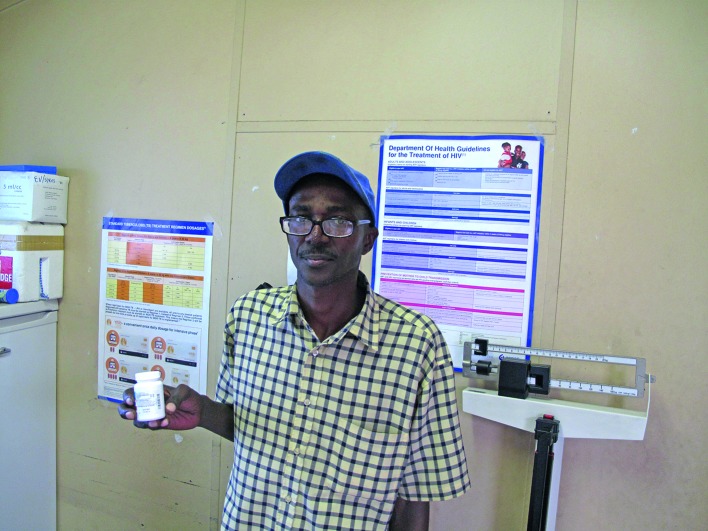
A man who has been on antiretroviral drugs for more than 10 years, and is now on third-line treatment, visits the Ubuntu Clinic in Khayelitsha, Cape Town, South Africa

WHO/T Namjilsuren (2013)

When the consolidated HIV guidelines document was being disseminated last year, WHO held regional workshops at which Easterbrook and her colleagues from the HIV department at WHO headquarters in Geneva mapped treatment policies that countries had implemented or were planning to implement.

An evaluation by the HIV department and others published in a supplement of the journal *AIDS* in March showed that, by November 2013, 68 countries were planning to bring their policies into line with the new treatment recommendation to change the threshold, so that they would initiate ARV treatment for patients with a CD4+ count of 500 cells/microlitre or less, irrespective of clinical status – a major change from the previous guidelines that recommended starting treatment for a CD4+ count of 350 cells/microlitre or less.

Increasingly seeking public input into the development of guidelines, WHO has started opening up its guidelines to public consultation. For example, in March WHO invited comments on revised sugar guidelines.

“The breadth and depth of the scope of WHO guidelines means that one size does not fit all, and some of the well-established methods may not be easily applicable to some subjects,” Penn says, adding that, ultimately, “the aim is to adhere to the core principles of best use of evidence, objectivity, freedom from bias, transparency and usefulness to the end user.”

